# A tumor cell specific Zona Pellucida glycoprotein 3 RNA transcript encodes an intracellular cancer antigen

**DOI:** 10.3389/fonc.2023.1233039

**Published:** 2023-12-06

**Authors:** Iman J. Schultz, Yvette Zimmerman, Cathy B. Moelans, Marcin Chrusciel, Jan Krijgh, Paul J. van Diest, Ilpo T. Huhtaniemi, Herjan J. T. Coelingh Bennink

**Affiliations:** ^1^Pantarhei Oncology BV, Zeist, Netherlands; ^2^Department of Pathology, University Medical Center Utrecht, Utrecht, Netherlands; ^3^Institute of Biomedicine, University of Turku, Turku, Finland; ^4^Institute of Reproductive and Developmental Biology, Imperial College London, London, United Kingdom

**Keywords:** ZP3, RNA transcript, intracellular, cancer antigen, oocyte

## Abstract

**Background:**

Expression of Zona Pellucida glycoprotein 3 (ZP3) in healthy tissue is restricted to the extracellular Zona Pellucida layer surrounding oocytes of ovarian follicles and to specific cells of the spermatogenic lineage. Ectopic expression of ZP3 has been observed in various types of cancer, rendering it a possible therapeutic target.

**Methods:**

To support its validity as therapeutic target, we extended the cancer related data by investigating ZP3 expression using immunohistochemistry (IHC) of tumor biopsies. We performed a ZP3 transcript specific analysis of publicly available RNA-sequencing (RNA-seq) data of cancer cell lines (CCLs) and tumor and normal tissues, and validated expression data by independent computational analysis and real-time quantitative PCR (qPCR). A correlation between the ZP3 expression level and pathological and clinical parameters was also investigated.

**Results:**

IHC data for several cancer types showed abundant ZP3 protein staining, which was confined to the cytoplasm, contradicting the extracellular protein localization in oocytes. We noticed that an alternative ZP3 RNA transcript, which we term ‘ZP3-Cancer’, was annotated in gene databases that lacks the genetic information encoding the N-terminal signal peptide that governs entry into the secretory pathway. This explains the intracellular localization of ZP3 in tumor cells. Analysis of publicly available RNA-seq data of 1339 cancer cell lines (CCLs), 10386 tumor tissues (The Cancer Genome Atlas) and 7481 healthy tissues (Genotype-Tissue Expression) indicated that ZP3-Cancer is the dominant ZP3 RNA transcript in tumor cells and is highly enriched in many cancer types, particularly in rectal, ovarian, colorectal, prostate, lung and breast cancer. Expression of ZP3-Cancer in tumor cells was confirmed by qPCR. Higher levels of the ZP3-Cancer transcript were associated with more aggressive tumors and worse survival of patients with various types of cancer.

**Conclusion:**

The cancer-restricted expression of ZP3-Cancer renders it an attractive tumor antigen for the development of a therapeutic cancer vaccine, particularly using mRNA expression technologies.

## 1 Introduction

The zona pellucida (ZP) is a multilayered extracellular matrix surrounding oocytes that in humans consists of the structurally related proteins ZP1-4 (1, 2). It performs several important roles during oogenesis, fertilization and survival of the fertilized egg during the early developmental stages. The four proteins that constitute the ZP share multiple domains, including a ZP domain important for polymerization during ZP formation, a furin cleavage site for release from the cell membrane, and a transmembrane domain. To ensure anchorage into the plasma membrane and extracellular secretion, ZP1-4 also contain an N-terminal signal peptide that is removed upon entry into the secretory pathway. Genetic aberrations that impair functionality of the ZP proteins and affect formation of the ZP are associated with reduced female fertility or infertility (3).

With respect to fertilization, an essential role for the Zona Pellucida glycoprotein 3 (ZP3) has been described for mediating the interaction of sperm with the ZP of the oocyte (4) and the entry into the egg by inducing the acrosome reaction in sperm (5). Attesting to this important role are data that showed that an immune response to a peptide derived from mouse ZP3 or to peptides derived from human ZP3, induced a contraceptive effect in female mice (6) or in a female primate species (7), respectively. In addition, mutations in human *ZP3* are involved in female infertility (8–12). Therefore, *ZP3* is required for normal ZP formation and fertilization.

While *ZP3* mRNA is absent in resting oocytes, it is highly expressed during oogenesis (13, 14). After ovulation, *ZP3* mRNA is degraded and hardly detectable. Transcriptional activation of *ZP3* is mediated by the folliculogenesis specific basic helix-loop-helix transcription factor FIGLA (15, 16). Long considered a gene solely expressed in developing oocytes, *ZP3* was recently shown to be expressed at both the mRNA and protein level in cells of the spermatogenic lineage (17). *ZP3* mRNA or protein was not detected in other normal human and mouse tissues (17). The physiological role of *ZP3* in spermatogenesis remains to be investigated and may not be related to fertility, as male mice homozygous null for *Zp3* reproduce normally (18, 19).

In addition to the expression in female and male gametes under physiological circumstances, ectopic expression of ZP3 has been shown in cancer (20–22). ZP3 protein was abundantly expressed in granulosa cell tumors, a rare form of ovarian cancer, and was unexpectedly shown to localize to the cytoplasm (20), contradicting the plasma membrane localization and extracellular secretion of ZP3 in oocytes. Also in the prostate cancer cell line PC3, ZP3 protein appeared dominantly cytoplasmic (21). Robinson et al. (22) analyzed transcriptome data of 32 cancer types from The Cancer Genome Atlas (TCGA) and of 30 healthy tissues, focusing on genes encoding secretory proteins. From a total of 1816 genes classified as secretory, a core set of 16 genes, which included *ZP3*, was identified of which the RNA expression was increased in most of the cancer types as compared to normal tissues. When the authors compared their set of 1816 secretory genes to a database containing experimentally validated extracellularly secreted proteins, the 800 genes that were found in common did not include *ZP3* (22). Another study investigated the secretion of proteins into the growth medium by a number of cancer cell lines (CCLs) (23). Most of these cell lines displayed high *ZP3* mRNA expression levels, but ZP3 protein was not detected in the medium. The findings from these different studies raised the question whether tumor cells produce an alternative ZP3 protein compared to that expressed in oocytes. While the enriched expression of *ZP3* in cancer versus normal tissue provides a therapeutic opportunity, whether ZP3 is secreted from cancer cells or not is an important strategic determinant for the development of a cancer immunotherapy based on active or passive immunization. The proof-of-principle for the development of a ZP3-based cancer vaccine for active immunization has been demonstrated in a transgenic mouse model of ovarian granulosa cell cancer (20).

In the present study, we aimed to broaden our view of the cellular localization pattern of ZP3 by investigating protein expression in different types of cancer. Localization of ZP3 protein in tumor cells appeared nearly exclusively cytoplasmic. An alternative *ZP3* RNA transcript that lacks the genetic information encoding the N-terminal signal peptide that governs entry into the secretory pathway was noted in gene databases. Analysis of publicly available RNA-sequencing data of CCLs, tumor tissues and healthy tissues revealed that this alternative *ZP3* mRNA transcript, which we term ZP3-Cancer, is dominantly expressed in tumor cells and highly enriched in various types of cancer. This classifies ZP3-Cancer largely as a tumor specific antigen and renders it a potential tumor marker and attractive immunotherapeutic target for the treatment of cancer.

## 2 Materials and methods

### 2.1 Human tissues

Redundant human formalin-fixed and paraffin-embedded tumor tissues (lung squamous cell/ovarian/breast/esophageal carcinoma) and normal ovarian tissue were obtained from the tissue bank of the University Medical Center (UMC) Utrecht (The Netherlands). Anonymous use of redundant tissue for research purposes is part of the standard treatment agreement with patients in the UMC Utrecht (24).

### 2.2 Immunohistochemistry

A monoclonal antibody (Isotype IgG1 kappa) specific to human ZP3 (a peptide with amino acid sequence RQPHVMSQWSRSASRN) was produced using the commercially available hybridoma from ATCC (ATCC^®^ CRL-2569™) as described previously (17). Formalin-fixed paraffin-embedded samples were deparaffinized and hydrated. Slides were boiled for 15 min in antigen retrieval buffer (10 mM citric acid buffer/0.05% Tween20, pH=6). To reduce non-specific background staining, sections were incubated in a humidified chamber for 1 h at room temperature (RT) with 3% BSA. Next, slides were incubated overnight in a humidified chamber at 4°C with the primary anti-human ZP3 antibody (1.0 μg/mL for tumor tissue, 0.125 μg/mL for ovarian tissue). Slides were then incubated in 0.5% H_2_O_2_ in PBS for 20min at RT to block endogenous peroxidase activity. DAKO polymer (DAKO EnVision+ System – HRP labelled polymer; Agilent, Santa Clara, CA, USA) was added to each slide and incubated in a humidified chamber for 30 min at RT, after which DAB+ Chromogen (DAKO) was applied for 5 min at RT. Slides were washed in dH_2_O, counterstained in Mayer’s hematoxylin (Sigma-Aldrich, Saint Louis, MO, USA), dehydrated and mounted with Pertex (Histolab Products, Askim, Sweden).

### 2.3 Cell culture

MDA-MB-453 (ATCC; breast cancer) cells were cultured in RPMI 1640 medium (Gibco, Thermo Fisher Scientific, Waltham, MA, USA) supplemented with 1% penicillin-streptomycin (v/v, Thermo Fisher Scientific) and 10% fetal bovine serum (v/v, Thermo Fisher Scientific). The cells were incubated at 37°C in a humidified atmosphere containing 5% CO_2_.

### 2.4 ZP3 transcript expression analysis and correlation with pathological and clinical parameters.

Publicly available *ZP3* transcript-level RNA-seq data of 1339 CCLs of the Cancer Cell Line Encyclopedia (CCLE; The Broad Institute) was downloaded from the DepMap portal (https://depmap.org/portal/; file ‘CCLE_RNAseq_transcripts.csv’, version 22Q2). This file contained expression data for seven *ZP3* RNA transcripts annotated in the Ensembl database (https://www.ensembl.org/index.html; **Table 1**). For 950 of the CCLs, an independent computational expression analysis for two of these *ZP3* transcripts (ENST00000336517/ZP3-Cancer, ENST00000394857/ZP3-Oocyte) was performed by Nebion AG (Zurich, Switzerland) based on curated CCLE data in their platform GENEVESTIGATOR^®^ (https://genevestigator.com) (25). A script was developed using a customized Python code (based on Python version 3.6.9) to distinguish between the two isoforms using the human reference transcriptome Ensembl 97, GRCh38.p12. The TPM (Transcripts Per Million) expression values were obtained by mapping libraries of reads to the reference transcriptome using Salmon (version 1.1, with default values for non-performance related parameters).

**Table 1:** Overview of annotated ZP3 RNA transcripts.

Ensemble Transcript ID	NCBI Refseq	Alternative name*	Protein Coding/Non-coding
ENST00000336517.8	NM_007155.6	ZP3-Cancer	Coding
ENST00000394857.8	NM_001110354.2	ZP3-Oocyte	Coding
ENST00000394860.3	–	ZP3-203	Coding
ENST00000416245.5	–	ZP3-204	Coding
ENST00000466960.5	–	ZP3-205	Non-coding
ENST00000467555.1	–	ZP3-206	Non-coding
ENST00000479793.5	–	ZP3-207	Non-coding

*ZP3-Cancer and ZP3-Oocyte are the ZP3 mRNA transcripts expressed in cancer and oocytes, respectively. The ZP3 names numbered 203-207 are from the Ensembl website.

*ZP3* transcript level RNAseq data for human cancer tissues and healthy human tissues were downloaded from the Genome Browser website of the University of California Santa Cruz Genomics Institute (https://genome.ucsc.edu/). The UCSC Computational Genomics lab quantified reads from publicly available RNAseq data for tumor tissues of The Genome Cancer Atlas (TCGA) consortium (https://www.cancer.gov/about-nci/organization/ccg/research/structural-genomics/tcga) and for normal tissues of the Genotype-Tissue Expression (GTEX) project (https://commonfund.nih.gov/GTEx) (26), and generated Transcript Per Million (TPM) expression values.

To investigate a correlation between ZP3-Cancer expression and pathological (tumor stage and grade) or clinical parameters [overall survival (OS); progression-free interval (PFI); disease-free interval (DFI); disease-specific survival (DSS)], previously curated pathological and clinical data for the tumors/patients of the TCGA were used (27). For survival analyses, data was initially uploaded to the online ‘Kaplan-Meier Plotter’ tool (https://kmplot.com/analysis/, ‘custom data’ option) using the ‘auto select best cutoff’ option, to explore whether any correlation existed between ZP3-Cancer expression level and the survival parameters. The resulting cut-offs were used to generate Kaplan-Meier survival curves in Graphpad Prism.

### 2.5 Real-time quantitative PCR

Total RNA of fresh frozen ovarian tissue confirmed to contain follicles was obtained from the tissue bank of the UMC Utrecht, The Netherlands, and was isolated using the AllPrep DNA/RNA/miRNA Universal Kit (Qiagen, Venlo, The Netherlands). RNA concentration and quality was determined by Nanodrop ND-2000 (Thermo Fisher Scientific). Total RNA extracted from the breast cancer cell line MCF7 (R1255830-50) and the cervical cancer cell line HeLa (R1255811-50) was purchased from Amsbio (Abingdon, UK). From MDA-MB-453, total RNA was isolated using the miRNeasy mini kit (Qiagen) according to the manufacturer’s instructions. Of all samples, one µg total RNA was used for cDNA synthesis in a single 20 µl reaction using the random primer-based High-Capacity cDNA Reverse Transcription Kit (Thermo Fisher Scientific) according to the manufacturer’s instructions. One µl cDNA (50 ng) was used in a single 20 µl SYBR Green-based qPCR reaction containing 10 µl iTaq Universal SYBR Green Supermix (2x; Bio-Rad) and 400 nM of forward and reverse primer. Each sample was analyzed in triplicate qPCR reactions. Primers were designed using NCBI Primer-BLAST and are indicated in **Table 2**. The ‘ZP3-both’ primer set detects the ZP3-Cancer and ZP3-Oocyte transcripts. *HPRT1* was used as reference gene for *ZP3* expression normalization. The real-time qPCR was performed on a ViiA7 system (Thermo Fisher Scientific). The PCR protocol was as follows: 15 sec at 95°C, 1 min at 60°C for a total of 40 cycles, followed by melting curve analysis by instrument default. Expression was calculated using the formula 2^-ΔCt^, where ΔCt is the Ct (Cycle threshold) of the target minus the Ct of the reference gene *HPRT1*.

**Table 2:** Primers used for real-time quantitative PCR.

Target	Primer	Primer sequence (5′-3′)	Amplicon size
ZP3-Both	Forward	TGTGAGCCTCTGGTCTCCAT	102
	Reverse	CACCAGGGCATCGTCAGTTA	
ZP3-Oocyte	Forward	TACTGAGCTGTGCTACCCCC	286
	Reverse	CACCAGGGCATCGTCAGTTA	
ZP3-Cancer	Forward	CTGTGCAGGATCTCCACTCG	107
	Reverse	CATGACCATCAGAGTGGCCT	
HPRT1	Forward	ACACTGGCAAAACAATGCAGA	99
	Reverse	TTCGTGGGGTCCTTTTCACC	

### 2.6 Statistical analysis

Statistical analyses were performed using Graphpad Prism. Pearson correlation analysis was performed to investigate a correlation in ZP3-Cancer expression between the Nebion and DepMap datasets for the cancer cell lines. The Mann-Whitney test (for comparing two groups) or the Kruskal-Wallis ANOVA test (for comparing three or more groups) were used to test differences in ZP3-Cancer transcript expression. For survival analyses, Kaplan-Meier curves were generated that were compared using the Log-rank (Mantel-Cox) test. A p-value <0.05 indicated statistical significance.

## 3 Results

### 3.1 ZP3 protein is expressed in cancer and is cytoplasmic

**Figure 1:**
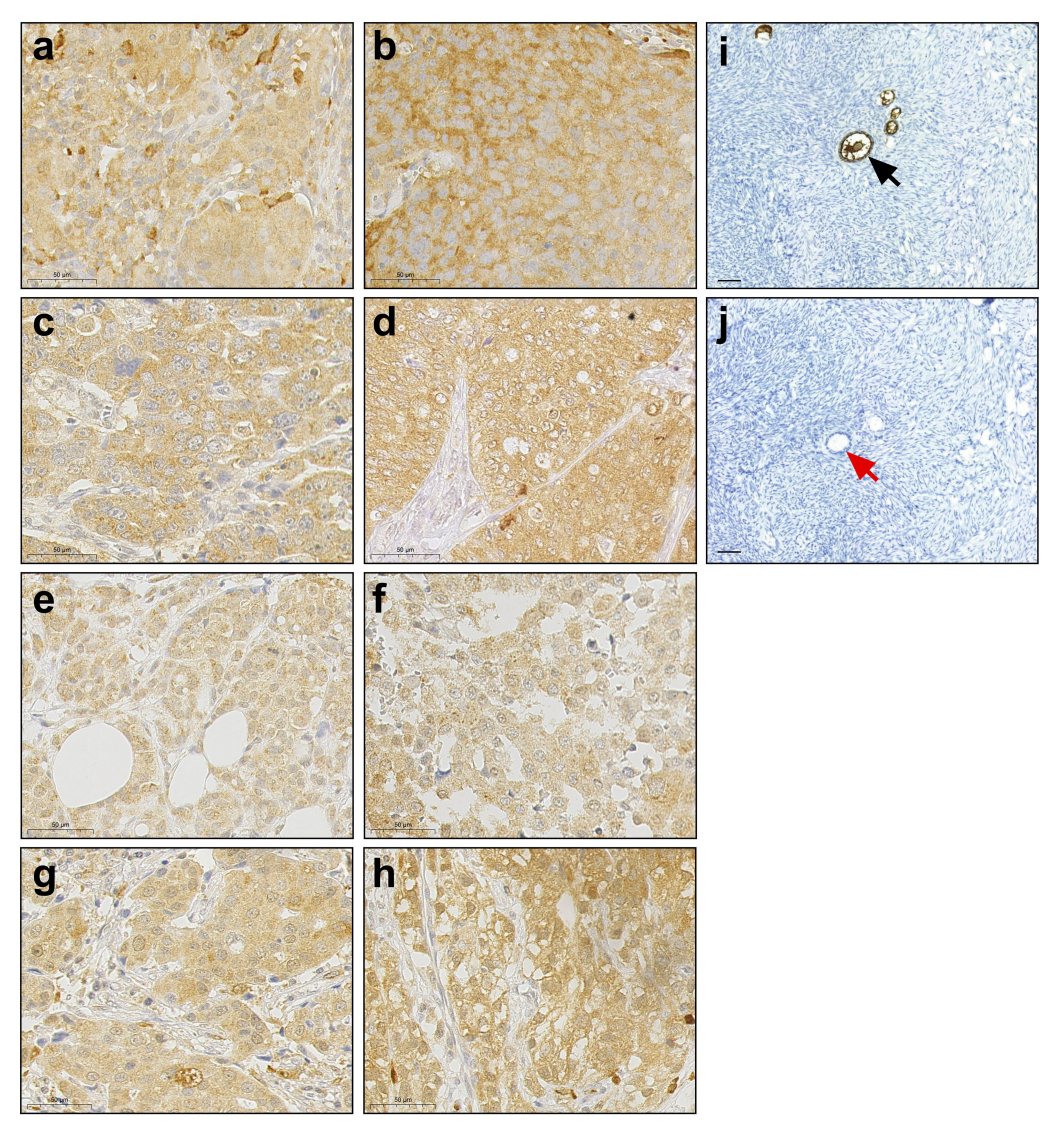
ZP3 protein is expressed in human cancer tissues and is cytoplasmic. Immunohistochemical detection of ZP3 protein in **(A, B)** lung squamous cell carcinoma; **(C, D)** ovarian adenocarcinoma; **(E)**, **(F)** breast carcinoma and **(G, H)** esophageal adenocarcinoma. Scale bar = 50 µm. **(I)** The ZP3 antibody specifically detects ZP3 in the zona pellucida of oocytes in ovarian follicles (indicated by black arrow), while no staining of oocytes in ovarian follicles is observed in the absence of the antibody (**J**; indicated by red arrow). Scale bar = 100 µm **(I, J)**.

To expand the picture of cancer related expression and cellular localization of ZP3, we performed IHC for a number of tumor tissues obtained from patients with different types of cancer (**Figure 1**). The monoclonal antibody we used has been raised against a peptide corresponding to amino acids 335-350 of human ZP3 (28). ZP3 protein was detected in lung squamous cell carcinoma, ovarian adenocarcinoma, breast carcinoma and esophageal adenocarcinoma. In all cases ZP3 was found to localize predominantly to the cytoplasm, with no obvious localization to the plasma membrane or to an extracellular layer (**Figures 1A-H**). This strongly contradicts the localization of ZP3 protein in (developing) oocytes (**Figure 1I**).

### 3.2 An alternative ZP3 transcript is dominantly expressed in cancer

**Figure 2:**
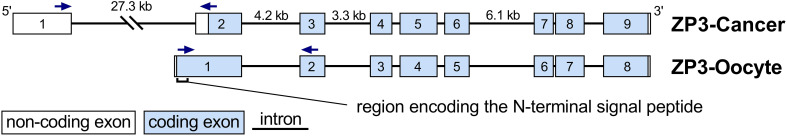
Exon-intron structures of the ZP3-Cancer and ZP3-Oocyte RNA transcripts. The ZP3-Cancer and ZP3-Oocyte RNA transcripts contain 9 and 8 exons respectively, of which exon 3-9 of ZP3-Cancer and 2-8 of ZP3-Oocyte are identical. The 5′-coding part of ZP3-Oocyte exon 1 encodes the N-terminal signal peptide that ensures entry of the ZP3 protein into the secretory pathway. This region is not part of exon 2 of the ZP3-Cancer transcript, and therefore is absent from the ZP3-Cancer mRNA. Exon 1 of ZP3-Cancer is located 27.3 kilobase pairs (kb) upstream of its second exon. Sizes (in kb) of the largest introns are indicated. The arrows indicate the locations of the primers used for transcript-specific detection by qPCR.

To find an explanation for the differential cellular localization of ZP3 in oocytes and tumor cells, we took a closer look at the *ZP3* mRNA isoforms annotated in the NCBI Genbank and Gencode/Ensembl databases. An mRNA variant lacking the genetic information encoding the first 51 amino acids of the oocyte ZP3 protein, which includes the N-terminal signal peptide (22 amino acids) governing entry into the secretory pathway, was noted in both databases (NM_007155.6/ENST00000336517.8). The exon-intron structures of the canonical *ZP3* transcript, ZP3-Oocyte, and the *ZP3* transcript we named ‘ZP3-Cancer’ are depicted in **Figure 2**. The first exon of the ZP3-Cancer transcript is located approximately 27 kilobase pairs upstream of its second exon, which largely overlaps with the first exon of the ZP3-Oocyte transcript, suggesting that a different transcription activation mechanism regulates ZP3-Cancer expression. In addition, expression of the transcription factor that activates *ZP3* expression in developing oocytes, FIGLA, was found to be virtually absent in CCLs (n=1355) and tumor tissues (n=11133) (median expression of 0.0 in both sample cohorts, data not shown). Therefore, the transcriptional activation of ZP3-Cancer appears to differ from that of ZP3 in oocytes.

**Figure 3:**
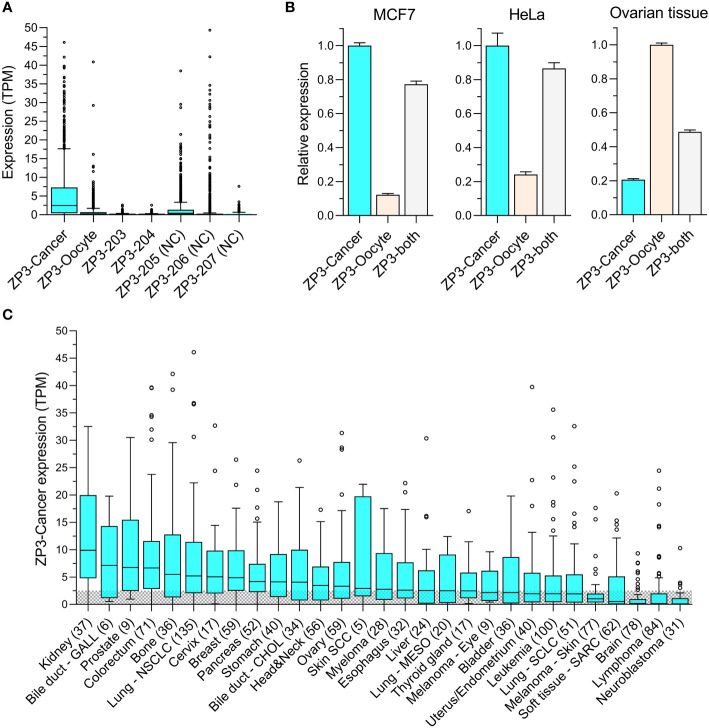
ZP3-Cancer is the dominant transcript in tumor cells and is differentially distributed among cancer types. **(A)** Expression of the seven annotated ZP3 transcripts in CCls (n=1339). Bars represent Tukey box plots (boxes are median+IQR). Median transcript expression levels (from left to right) are 2.47, 0.22, 0.0, 0.04, 0.42, 0.0 and 0.0 TPM. ZP3-Cancer expression is significantly higher compared to all other transcripts (Kruskal-Wallis test, p < 0.0001). NC = Non-protein coding. **(B)** Relative expression of ZP3-cancer and ZP3-Oocyte in the CCLs MCF7 and HeLa, and in healthy ovarian tissue containing follicles, as determined by qPCR. ZP3-Cancer expression is set to 1 for MCF7 and HeLa, ZP3-Oocyte expression is set to 1 for ovarian tissue. ‘ZP3-both’ served as a positive control and used primers designed to detect ZP3-Cancer and ZP3-Oocyte simultaneously. Bars represent mean+SD for the triplicate qPCR reactions. **(C)** Differential expression of ZP3-Cancer in the cancer types covered by the CCLs. Cancer types represented by less than 5 CCLs were not included. Bars represent Tukey box plots (boxes are median+IQR). Number of samples are indicated between brackets. The shaded area indicates CCLs with a ZP3-Cancer expression level below the overall median (2.54 TPM).

Publicly available RNAseq data for the seven annotated *ZP3* RNA transcripts of 1339 CCLs was analyzed to obtain insight into their expression level in tumor cells. Clearly, ZP3-Cancer is the dominant transcript (**Figure 3A**). Its median expression (2.47 TPM) is more than 10x higher compared to that of ZP3-Oocyte (0.22 TPM), whose expression is essentially absent, which also applies to the other two protein-coding transcripts (ZP3-203 and ZP3-204). Two of the three non-coding transcripts (ZP3-205 and ZP3-206) are only expressed at relatively high levels in a limited number of CCLs. An independent quantification of the transcript levels of ZP3-Cancer and ZP3-Oocyte in 950 overlapping CCLs confirms ZP3-Cancer is expressed at much higher levels (**Supplementary Figure S1A**), with a strong correlation in expression of these two transcripts between the two datasets attesting to the validity of the findings (R^2 =^ 0.99 and 0.94 for ZP3-Cancer and ZP3-Oocyte, resp.; **Supplementary Figures S1B, C**).

To confirm the existence of ZP3-Cancer in tumor cells, and compare its expression level with that of ZP3-Oocyte, we performed real-time qPCR for a number of CCLs and ovarian tissue using transcript specific primers (see **Figure 2** for primer localization). Two CCLs with high ZP3-Cancer expression according to the RNAseq data (MCF7 and HeLa, with respective ZP3-Cancer expression of 13.3 and 7.3 TPM, and respective ZP3-Oocyte expression of 0.42 and 1.0 TPM), one cell line with very low ZP3-Cancer expression (MDA-MB-453, 0.01 TPM, and ZP3-Oocyte expression of 0.0 TPM), and ovarian tissue were selected. The ZP3-Cancer transcript was detected in both MCF7 and HeLa cells, and at much higher levels compared to the ZP3-Oocyte transcript (**Figure 3B**), corroborating the RNAseq data. In contrast, in ovarian tissue, the ZP3-Oocyte transcript is more abundantly expressed (**Figure 3B**). ZP3-Cancer was not detected in MDA-MB-453 cells (data not shown).

Next, we investigated whether ZP3-Cancer was differentially expressed across the different cancer types covered by the CCLs. ZP3-Cancer transcript levels appear enriched in solid tissue type cancers, while those originating in the brain or are blood cell derived generally show low(er) expression levels (**Figure 3C**).

### 3.3 ZP3-Cancer is strongly enriched in tumor tissue

**Figure 4:**
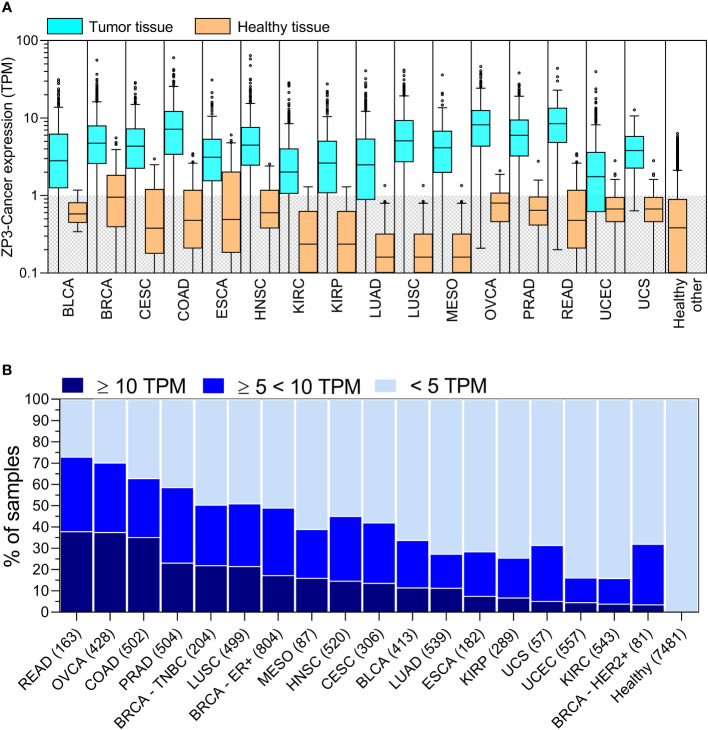
ZP3-Cancer expression is strongly enriched in tumor tissue and is cancer (sub)type enhanced. **(A)** ZP3-Cancer expression in tumor tissues relative to expression in respective healthy tissues (statistically significant upregulation, p < 0.0001 for all cancer types, Mann-Whitney test). Bars represent Tukey box plots (boxes are median+IQR). The shaded area marks tissues with very low ZP3-Cancer expression levels (< 1 TPM). BLCA, bladder urothelial carcinoma (n=413; healthy n=9); BRCA, breast invasive carcinoma (n=1132; healthy n=181); CESC, cervical squamous cell carcinoma (n=306; healthy n=10); COAD, colorectal adenocarcinoma (n=502; healthy n=308); ESCA, esophageal carcinoma (182; healthy n=655); HNSC, head-neck squamous cell carcinoma (n=520; healthy n=55); KIRC, kidney renal clear cell carcinoma (n=543; healthy n=28); KIRP, kidney renal papillary cell carcinoma (n=289; healthy n=28); LUAD, lung adenocarcinoma (n=539; healthy n=295); LUSC, lung squamous cell carcinoma (n=501; healthy n=295); MESO, mesothelioma (n=87; healthy n=295); OVCA, ovarian carcinoma (n=428; healthy n=88); PRAD, prostate adenocarcinoma (n=504; healthy n=100); READ, rectal adenocarcinoma (n=163; healthy n=308); UCEC, uterine corpus endometrial carcinoma (n=557; healthy n=79); UCS, uterine carcinosarcoma (n=57; healthy n=79); Healthy other n=5673. The same healthy tissue cohorts were used for COAD and READ (n=308), KIRC and KIRP (n=28), LUAD, LUSC and MESO (n=295), and UCEC and UCS (n=79). **(B)** ZP3-Cancer expression level distribution among the different cancer (sub)types. Tumors were categorized according to three expression level categories, with a stringent cut-off of 5 TPM for low expression. Cancer (sub)types are arranged in descending order of the % of samples with highest ZP3-Cancer expression (> 10 TPM). BRCA-TNBC, triple negative breast invasive carcinoma; BRCA-ER+, estrogen receptor positive breast invasive carcinoma; BRCA-HER2+, human epidermal growth factor receptor 2 positive (amplified) breast invasive carcinoma. The number of samples are indicated between brackets.

To obtain further insight into the cancer related expression of ZP3-Cancer, we analyzed publicly available, transcript-level RNAseq data for tumor tissues of the TCGA (10386 tumor tissues). In tumor tissues, ZP3-Cancer is also clearly the dominant protein-coding transcript (**Supplementary Figure S2A**). Next, we investigated whether ZP3-Cancer is selectively enriched in tumor cells relative to healthy tissue. A previous study showed that transcriptome profiles of tissue adjacent to tumors (‘adjacent normal tissue’) are different from those of healthy tissues (included in the GTEx project) and constitute a distinct intermediate group between healthy tissues and tumor tissues (29). The authors concluded that for differential expression analysis, healthy tissues provides a more accurate comparator compared to adjacent normal tissue. We noticed that ZP3-Cancer expression in normal tissue adjacent to tumors is significantly upregulated relative to healthy tissue, and is intermediately expressed in between healthy and tumor tissue (**Supplementary Figures S2B, C**). Therefore, we decided to compare ZP3-Cancer expression in tumor tissue to that in healthy tissue. ZP3-Cancer appeared highly significantly enriched in numerous cancer types as compared to the respective healthy tissues (p < 0.0001), but also to healthy tissues in general (**Figure 4A**). Among these cancer-types with enriched expression, the ZP3-Cancer expression level varied considerably (**Figure 4B**). To categorize tumors with respect to their ZP3-Cancer expression levels, we chose a stringent limit for low expression of 5 TPM, as this was the upper limit of ZP3-Cancer expression in healthy tissue (99.8% of healthy samples displayed a ZP3-Cancer expression level below 5 TPM, and 99.1% a level below 4 TPM). Patients with rectal adenocarcinoma (READ), ovarian carcinoma (OVCA) and colorectal adenocarcinoma (COAD) had the largest number of tumors with very high ZP3-Cancer expression (> 10 TPM), with 38.0, 37.6 and 35.3% of tumors in this category, respectively. For these cancer types, overall between 63% (COAD) and 73% (READ) of tumors display medium to high ZP3-Cancer expression (higher than 5 TPM). For patients with prostate adenocarcinoma (PRAD), triple-negative breast cancer (BRCA-TNBC) and lung squamous cell carcinoma (LUSC), at least 50% of tumors display medium to high ZP3-Cancer expression levels. Other types of cancer display lower numbers of tumors with medium to high expression, but still a significant percentage of tumors with ZP3-Cancer levels above those in healthy tissue (**Figure 4B**). Overall, the data show that ZP3-Cancer expression is enriched in cancer, and that its expression level varies considerably among the different cancer types.

### 3.4 ZP3-Cancer expression correlates with pathological and clinical parameters

**Figure 5:**
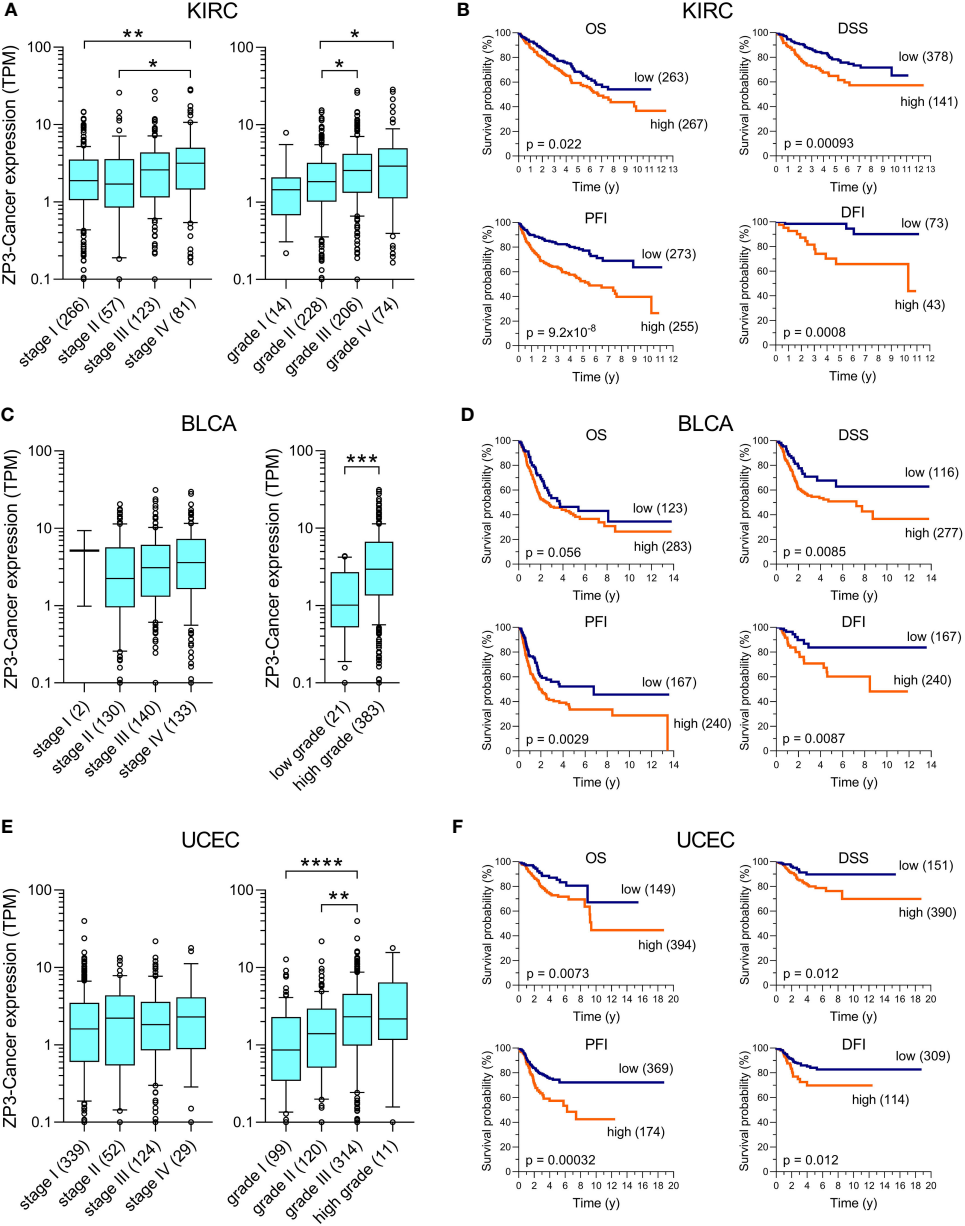
ZP3-Cancer expression correlates with pathological and clinical parameters in KIRC, BLCA and UCEC. Correlation of ZP3-Cancer expression level with tumor stage and grade in **(A)** KIRC, **(C)** BLCA and **(E)** UCEC, and with the survival parameters OS, DSS, PFI and DFI in **(B)** KIRC, **(D)** BLCA and **(F)** UCEC. **(A, C, E)** Bars represent Tukey box plots (boxes are median+IQR). The number of samples are indicated between brackets. Differences in ZP3-Cancer expression level between tumor stages and grades were determined using the Kruskal-Wallis test, except for BLCA tumor grade [**(C)**, Mann-Whitney test]. No difference in ZP3-Cancer expression was observed between tumor stages for BLCA **(C)** and UCEC **(E)**. *p < 0.05; **p < 0.01; ***p < 0.001; ****p < 0.0001. **(B, D, F)** Kaplan-Meier survival curves for OS, DSS, PFI and DFI in patients with **(B)** KIRC, **(D)** BLCA and **(F)** UCEC. Patients were separated into those with tumors with low or high ZP3-Cancer expression level (number of patients per group indicated between brackets), using the cut-off value obtained from Kaplan-Meier Plotter. Curves were compared using the Log-rank test. Time is in years (y).

To obtain an initial insight into the oncogenic characteristics of ZP3-Cancer, curated pathological (tumor stage and grade) and clinical (OS, PFI, DFI, DSS) data for the tumors and patients included in the TCGA were used to explore a relationship between these parameters and the ZP3-Cancer expression level. In kidney renal clear cell carcinoma (KIRC), uterine corpus endometrial carcinoma (UCEC) and bladder urothelial carcinoma (BLCA), more aggressive higher grade tumors displayed statistically elevated ZP3-Cancer transcript levels, while such a relationship was observed for advanced tumor stage only in KIRC (**Figures 5A, C, E**). In addition, an increased ZP3-Cancer expression level was statistically significantly associated with poorer outcome for all four distinct clinical survival parameters in KIRC and UCEC, and three of the four in BLCA (**Figures 5B, D, F**). We also observed statistically significant relationships between the ZP3-Cancer expression level and tumor grade and the clinical parameters OS and DSS in head and neck squamous cell carcinoma (HNSC; **Supplementary Figure S3A**), tumor grade and OS and DSS in pancreatic ductal adenocarcinoma (PAAD; **Supplementary Figure S3B**), and tumor stage and OS in lung adenocarcinoma (LUAD; **Supplementary Figure S3C**). In other cancer types, ZP3-Cancer expression did not show a relationship with pathological or clinical parameters (data not shown).

## 4 Discussion

In this study we describe the expression of a novel *ZP3* mRNA transcript in cancer cells that is different from the *ZP3* transcript expressed in oocytes. This novel transcript, which we termed ZP3-Cancer, lacks the genetic information encoding the N-terminal signal peptide, which, in oocytes, governs entry of the ZP3 protein into the secretory pathway, anchoring into the plasma membrane and eventually extracellular release into the ZP layer. Hence, the protein encoded by ZP3-Cancer in tumor cells remains cytoplasmic. Such ectopic expression of genes involved in normal physiology is observed frequently in cancer (30), for example genes expressed in normal testicular germ cells (31). It may be the consequence of aberrant expression of transcription factors (32). The latter seems relevant for ZP3-Cancer, as the key transcription factor responsible for *ZP3* expression in oocytes, *FIGLA*, appears not to be expressed in cancer.

Cytoplasmic expression of the ZP3 protein in cancer has been shown before in ovarian granulosa cell tumors (20), a rare form of ovarian cancer. In that study, intracellular ZP3 protein was also detected in the human serous ovarian cancer derived cell line OVCAR3. Another study showed ZP3 protein localization to the cytoplasm of the human prostate cancer cell line PC3, but there also appeared to be ZP3 staining at the cell membrane (21). According to the publicly available RNAseq data for CCLs, ZP3-Cancer is expressed in PC3 (4.8 TPM), but ZP3-Oocyte transcript levels are very low (0.6 TPM), while the other two ZP3 protein-coding transcripts (ZP3-203/-204), which also lack the genetic information for the N-terminal signal peptide, are absent. For the detection of *ZP3* transcripts by PCR, both of the aforementioned studies used primers that did not distinguish between ZP3-Cancer and ZP3-Oocyte. Data on the differential expression of these two transcripts would have supported the interpretation of the results.

The publicly available RNAseq data of CCLs and tumor tissues, and our independent computational and qPCR analysis, provide a clear explanation for the cytoplasmic localization of ZP3 observed in cancer, as these data show that ZP3-Cancer is the dominant transcript in tumor cells. The other two protein-coding *ZP3* transcripts that encode putative cytoplasmic proteins, ZP3-203 and ZP3-204, are expressed at very low levels in tumor cells, and are therefore unlikely to contribute to the cytoplasmic levels of ZP3 protein in cancer. Our findings also provide an explanation for the absence of ZP3 protein in a database of experimentally validated extracellularly secreted proteins and in the growth medium of CCLs with high ZP3 transcript expression levels (22, 23). Localization of ZP3 to the plasma membrane of tumor cells may be observed, as RNAseq analysis identified the ZP3-Oocyte transcript in cancer, but only in a very limited number of cases. To further define the localization of ZP3 protein in cancer cells, more detailed microscopic analysis is needed.

Our data show that ZP3-Cancer is expressed at varying levels in cancer (sub)types, but also that it is highly enriched in tumor tissues as compared to healthy tissues, where it is virtually absent. Cancer-enriched expression of ZP3 has been shown before (also a comparison of TCGA tumors and GTEx healthy tissues), although those findings related to ZP3 gene-level expression analysis (expression levels of all seven ZP3 transcripts combined) (22). Our ZP3 transcript-focused analysis of the same data cohorts strongly suggests that ZP3-Cancer dominates the cancer-enriched signature. It should be noted that publicly available RNAseq data suggests *ZP3* to be expressed in healthy tissues, but this can be largely ascribed to expression of the non-protein coding *ZP3* transcripts, and not to *ZP3* protein-coding transcripts [GTEx data portal for *ZP3* isoform expression, and our analysis of the UCSC transcript level data for the GTEx samples (data not shown)]. Therefore, for *ZP3* expression analysis it is important to segregate the data into individual transcript levels. In this respect, the near absence of the ZP3-Cancer transcript (or the other protein-coding *ZP3* transcripts) in healthy tissue is corroborated by recently published ZP3 IHC data for 14 different healthy human tissues and organs, showing the absence of protein expression (17). This study employed the same ZP3 antibody as used in our report, which detects not only the ZP3 proteins encoded by the ZP3-Cancer and ZP3-Oocyte transcripts, but also those encoded by the other two protein-coding transcripts (should they be translated). The only organs in which ZP3 protein was detected were the ovary (follicles/oocytes) and testis (cells of the spermatogenic lineage) (17). Another study performed a paired deep proteome and RNAseq analysis of twenty-nine healthy human tissues covering all major organs, and did not detect ZP3 protein among 13,640 identified proteins, while very low ZP3 transcript numbers (for the four protein-coding transcripts combined) were found in all tissues except heart (33). These data for healthy tissues, combined with the substantial enrichment of ZP3-Cancer in a number of cancer (sub)types, provides a promising therapeutic window.

To obtain a first glimpse of the oncological character of ZP3-Cancer, we investigated a relationship between its expression level and pathological and clinical parameters. This showed that increased ZP3-Cancer transcript levels are associated with more aggressive tumor cell characteristics and worse survival in a number of cancer types. An earlier study focusing on prognostic markers for renal clear cell carcinoma (KIRC; TCGA cohort) revealed significant correlations between ZP3 expression level and OS, DSS and progression-free survival (34). This study used ZP3 gene-level expression and did not differentiate between the individual ZP3 transcripts. As we found significant relationships between ZP3-Cancer transcript levels and OS, DSS and PFI in KIRC, the overlap in these data between the two studies may be explained by ZP3-Cancer being the dominant transcript of the gene-level ZP3 expression level in KIRC tumors. On the other hand, we also found a highly significant association between ZP3-Cancer expression and the DFI in KIRC patients, while Ahluwalia et al. did not (Log-rank p-value = 0.49). While the focus on the ZP3-Cancer transcript may be an explanation for the difference, it could also be due to the use of a different clinical dataset. Altogether, our data indicates that ZP3-Cancer may be involved in supporting tumor cell survival and aggressive behavior. Investigation of additional cohorts of cancer patients should show whether the significant association of ZP3-Cancer with clinical parameters can be reproduced and extended to other types of cancer. Cell-based phenotypic and genetic perturbation studies are needed to characterize the oncological properties of ZP3-Cancer in more detail.

## 5 Conclusions

The data presented here establish a new paradigm in the biology of ZP3, a protein thus far considered solely involved in human reproduction. The identification of ZP3-Cancer as an alternative *ZP3* transcript that encodes a protein localized to the cytoplasm of tumor cells, will spark research into its ectopic function. An initial interrogation of its oncological properties suggests ZP3-Cancer is associated with more aggressive behavior of tumor cells and may confer a survival advantage to these cells when expressed at higher levels. Our findings pinpoint ZP3-Cancer as a strongly cancer-enriched antigen, which provides a promising window for an immunotherapy-based intervention employing its unique mRNA sequence.

## Data availability statement

The datasets presented in this study can be found in online repositories. The names of the repository/repositories and accession number(s) can be found in the article/**Supplementary Material**.

## Ethics statement

Ethical approval was not required for the studies involving humans because See reference in the paper stating that the human tumor material that was used in this study did not need ethical approval. The studies were conducted in accordance with the local legislation and institutional requirements. The human samples used in this study were acquired from a by- product of routine care or industry. Written informed consent to participate in this study was not required from the participants or the participants’ legal guardians/next of kin in accordance with the national legislation and the institutional requirements.

## Author contributions

Conceptualization: IS, YZ, JK, PD, IH and HC; Data acquisition: IS, YZ, CM, MC and PD; Data analysis: IS, YZ, CM, MC, PD and IH; Supervision: IS, JK, PD, IH and HC; Writing of the original draft: IS; All authors contributed to the article and approved the submitted version.
