# Supplementary material for: A tumor cell specific Zona Pellucida glycoprotein 3 RNA transcript encodes an intracellular cancer antigen
Source: Front Oncol. 2023 Dec 6;13:1233039. doi: 10.3389/fonc.2023.1233039 (PMC10731367; doi:10.3389/fonc.2023.1233039)
Supplement: Supplementary file 1 [file DataSheet_1.pdf]

frontiers

Supplementary Material

A tumor cell specific Zona Pellucida glycoprotein 3 RNA transcript encodes an intracellular cancer antigen

Iman J. Schultz, Yvette Zimmerman, Cathy B. Moelans, Marcin Chrusciel, Jan Krijgh, Paul J. van Diest, Ilpo T. Huhtaniemi, Herjan J. T. Coelingh Bennink*

*** Correspondence:** Corresponding Author: hcb@pantarheibio.com

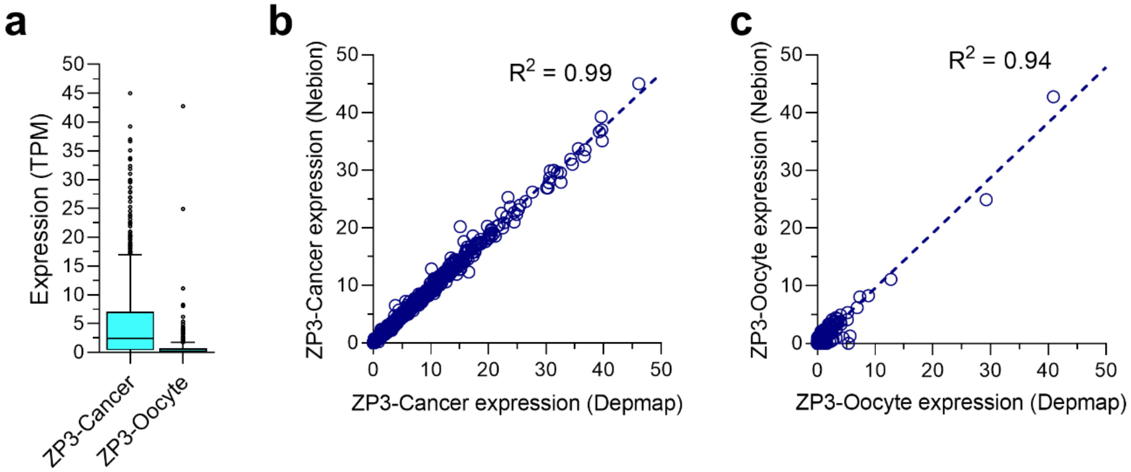

Supplementary Figure S1. Independent quantification of ZP3-Cancer and ZP3-Oocyte expression in CCLs. (**a**) ZP3-Cancer and ZP3-Oocyte expression in CCls (n=950), as determined by Nebion. Bars represent Tukey box plots (boxes are median+IQR). Median ZP3-Cancer and ZP3-Oocyte expression are 2.42 and 0.17 TPM, respectively. Pearson correlation between (**b**) ZP3-Cancer expression determined in the 950 CCLs by the Depmap consortium and Nebion, and (**c**) ZP3-Oocyte expression determined in the 950 CCLs by the Depmap consortium and Nebion.

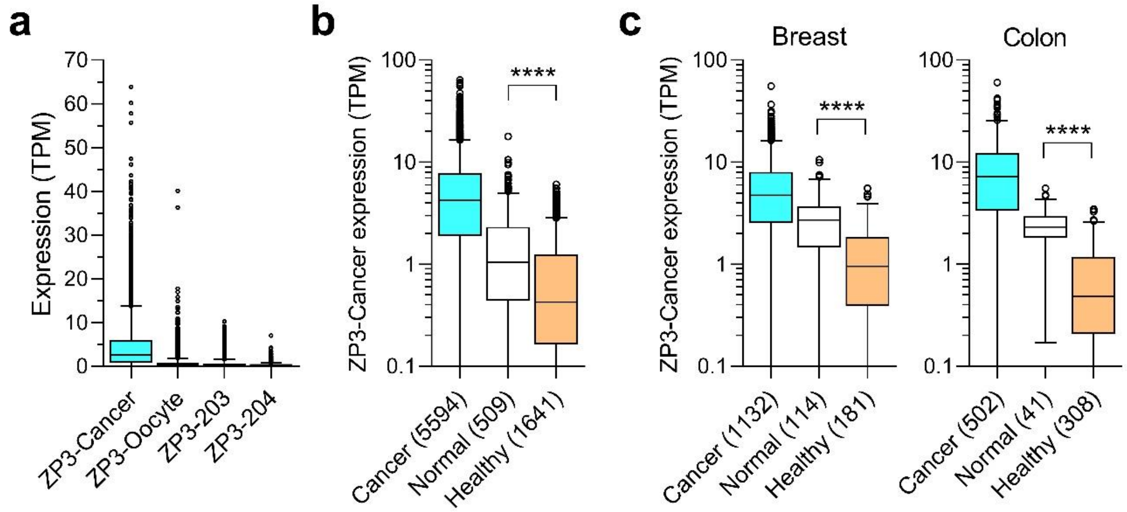

Supplementary Figure S2. ZP3-Cancer is the dominant ZP3 transcript in tumor tissues and is upregulated in normal tissue adjacent to tumors. (**a**) Expression of the ZP3 protein coding transcripts in tumor tissues (n=10386). Bars represent Tukey box plots (boxes are median+IQR). Median expression levels of the four transcripts are (from left to right), 2.59, 0.23, 0.087 and 0.27 TPM. ZP3-Cancer expression in (**b**) tumor tissues ('Cancer'), normal tissue adjacent to tumors ('Normal') and healthy tissue, and, separately, in (**c**) cancer, normal and healthy tissues of the breast and colon. (**b**) Normal and healthy samples included are those accompanying the cancer types presented in Figure 4a (cancer types for which no Normal samples were available were excluded, as well as their respective healthy tissue samples). (**b**), (**c**), Bars represent Tukey box plots (boxes are median+IQR; number of samples indicated between brackets). The ZP3-Cancer expression levels in Normal and Healthy samples were compared using the Mann-Whitney test (****, $p < 0.0001$).

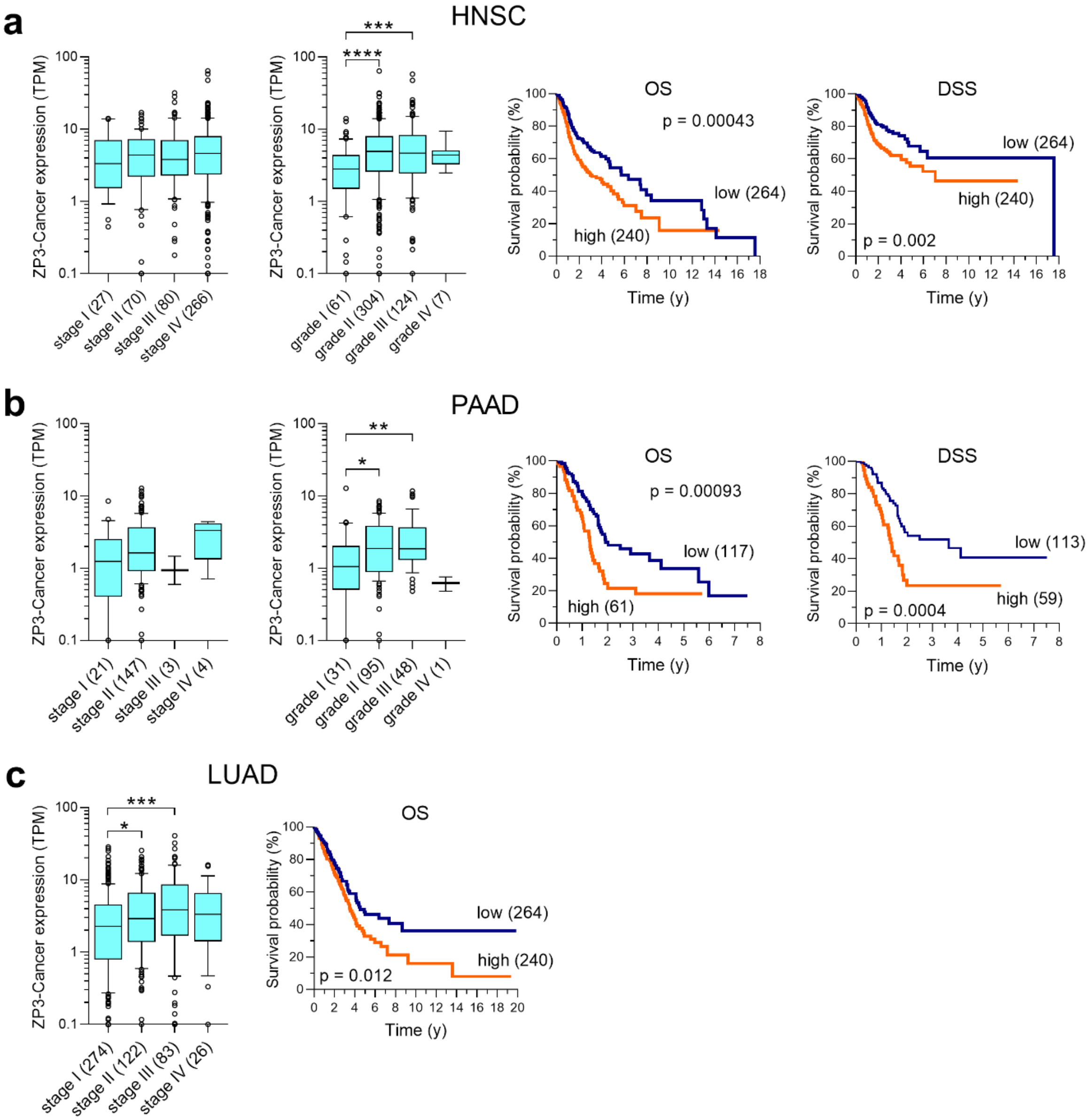

Supplementary Figure S3. ZP3-Cancer expression correlates with pathological and clinical parameters in HNSC, PAAD and LUAD. Correlation of ZP3-Cancer expression level with tumor stage, tumor grade and the survival parameters OS and DSS in (**a**) HNSC (head-neck squamous cell carcinoma), (**b**) PAAD (pancreatic ductal adenocarcinoma) and (**c**) LUAD (lung adenocarcinoma). Bars for tumor stage and grade represent Tukey box plots (boxes are median+IQR; number of samples indicated between brackets). Differences in ZP3-Cancer expression level between tumor stages and grades were determined using the Kruskal-Wallis test (*, $p < 0.05$; **, $p < 0.01$; ***, $p < 0.001$; ****, $p < 0.0001$). For Kaplan-Meier survival analysis of OS and DSS, patients were separated into those with tumors with low or high ZP3-Cancer expression level (number of patients per group indicated between brackets), using the cut-off value obtained from Kaplan-Meier Plotter. Curves were compared using the Log-rank test. Time is in years (y).
